# Impact of interictal epileptiform discharges on brain network in self‐limited epilepsy with centrotemporal spikes: A magnetoencephalography study

**DOI:** 10.1002/brb3.3038

**Published:** 2023-05-03

**Authors:** Yue Xu, Yingfan Wang, Fengyuan Xu, Yihan Li, Jintao Sun, Kai Niu, Pengfei Wang, Yanzhang Li, Ke Zhang, Di Wu, Qiqi Chen, Xiaoshan Wang

**Affiliations:** ^1^ Department of Neurology The Affiliated Brain Hospital Nanjing Medical University Nanjing Jiangsu P. R. China; ^2^ MEG Center Nanjing Brain Hospital Nanjing Jiangsu P. R. China

**Keywords:** functional connectivity, high‐frequency oscillations, interictal epileptiform discharges, magnetoencephalography, self‐limited epilepsy with centrotemporal spikes

## Abstract

**Objective:**

This study aimed to investigate the differences on resting‐state brain networks between the interictal epileptiform discharge (IED) group with self‐limited epilepsy with centrotemporal spikes (SeLECTS), the non‐IED group with SeLECTS, and the healthy control (HC) group.

**Methods:**

Patients were divided into the IED and non‐IED group according to the presence or absence of IED during magnetoencephalography (MEG). We used Wechsler Intelligence Scale for Children, fourth edition (WISC‐IV) to assess cognition in 30 children with SeLECTS and 15 HCs. Functional networks were constructed at the whole‐brain level and graph theory (GT) analysis was used to quantify the topology of the brain network.

**Results:**

The IED group had the lowest cognitive function scores, followed by the non‐IED group and then HCs. Our MEG results showed that the IED group had more dispersed functional connectivity (FC) in the 4–8 Hz frequency band, and more brain regions were involved compared to the other two groups. Furthermore, the IED group had fewer FC between the anterior and posterior brain regions in the 12–30 Hz frequency band. Both the IED group and the non‐IED group had fewer FC between the anterior and posterior brain regions in the 80–250 Hz frequency band compared to the HC group. GT analysis showed that the IED group had a higher clustering coefficient compared to the HC group and a higher degree compared to the non‐IED group in the 80–250 Hz frequency band. The non‐IED group had a lower path length in the 30–80 Hz frequency band compared to the HC group.

**Conclusions:**

The study data obtained in this study suggested that intrinsic neural activity was frequency‐dependent and that FC networks of the IED group and the non‐IED group underwent changes in different frequency bands. These network‐related changes may contribute to cognitive dysfunction in children with SeLECTS.

## Graphical abstract

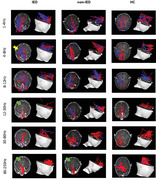

## 1 INTRODUCTION

Self‐limited epilepsy with centrotemporal spikes (SeLECTS), also known as Rolandic epilepsy, is the most common idiopathic focal epilepsy syndrome in children, accounting for approximately 15–20% of seizures in children under 15 years of age (Panayiotopoulos et al., 2008; Wirrell, 1998). Typical symptoms of this type of epilepsy include local motion‐sensing seizures in the mouth and one side of the face, which can be extended to generalized tonic‐clonic seizures (Archer et al., 2003). In the past, SeLECTS was considered a self‐limited disease with a good prognosis and complete remission during puberty (Li et al., 2020). However, recent studies have revealed that neuropsychological disorders are common in children with SeLECTS, and include poor performance in processing speed, fine motor skills, and long‐term storage (Li et al., 2020; Niu et al., 2021; Vannest et al., 2016). Moreover, SeLECTS patients are more likely to have emotional problems such as anxiety and depression (Liu & Han, 2016).

The typical pattern of electroencephalography (EEG) is the presence of stereotyped high‐voltage blunt spike‐slow wave discharges in the central temporal region, especially during sleep (Riva et al., 2007; Wickens et al., 2017). Interictal epileptiform discharges (IED) have been shown to cause alterations in brain networks. IED may directly lead to transient suppression of cognition‐related networks. The default mode network was selectively impaired during IED (Li et al., 2017). Moreover, IED disrupted the language networks, leading to impaired perceptual processing and expression of language, affecting literacy and thus leading to dyslexia (Chen et al., 2018; Xiao et al., 2016). It also resulted in reduced connectivity within the dorsal attention network, thereby impairing attention and executive abilities (Li et al., 2017). Chronic epileptogenic processes may lead to aberrant functional neural circuit organization, contributing to neurocognitive function impairment in patients with SeLECTS (Li et al., 2017). Frequent IED is a potential risk factor for learning and behavioral difficulties, reduced language comprehension and expression, and poor performance on neuropsychological tests (Riva et al., 2007; Volkl‐Kernstock et al., 2009). However, the relationship between epileptic discharges and functional abnormalities, and cognitive function has not been thoroughly assessed in patients with SeLECTS.

Now, many noninvasive examinations of the brain have been applied to study functional connectivity (FC) networks. Magnetoencephalography (MEG) has a high time resolution and allows for the analysis of brain network changes in multiple frequency bands (Sun et al., 2021b; Sun et al., 2020a; Wu et al., 2016). Therefore, MEG has been used in the study of brain networks in many diseases including epilepsy, schizophrenia, and migraine (Wu et al., 2016; Niu et al., 2021). Previous MEG studies found that large‐scale changes in FC were observed before, during, and after the onset of IED (Ibrahim et al., 2014). A study about neuromagnetic activities found differences in source location between the IED, the non‐IED, and the HC groups at low frequency band (<80 Hz). They also observed that the strength of gamma oscillations in the chronic epilepsy state reflected the duration of SeLECTS. Wang et al. found that, compared with the HC group, SeLECTS patients showed increased frontal cortex connections in specific frequency bands. Moreover, children with benign epilepsy with bilateral centrotemporal spikes showed a more disorderly and randomized network in the 1–4 and 80–250 Hz frequency bands. The aim of this study was to investigate differences in networks between the IED group, the non‐IED group in patients with SeLECTS, and the healthy controls (HC) using MEG and explore the relationship between epileptic discharges, network abnormalities, and cognitive function.

## 2 EXPERIMENTAL PROCEDURES

### 2.1 Subjects

A total of 35 children aged 6–13 years with confirmed SeLECTS were recruited from the Neurology Department of the Children's Hospital of Nanjing Medical University and the Nanjing Brain Hospital, whose diagnoses met the seizure criteria of the ILAE classification. Out of the 35 diagnosed patients with SeLECTS, 3 had a history of febrile convulsions and 2 had a history of encephalitis; thus, only 30 patients were recruited for this study. In addition, 20 healthy children without any history of neuropsychiatric disorders and other major diseases and whose intelligence levels were in the normal range were recruited. Finally, 15 healthy children matching the SeLECTS group in terms of age, sex, family background, and economic income were selected. Based on the presence of IED during MEG, patients were divided into two groups: the IED group and the non‐IED group. Clinical data of patients are listed in Table 1. The inclusion criteria were as follows: (1) a diagnosis of SeLECTS according to the ILAE 2017 classification of epilepsy syndrome (Scheffer et al., 2017); (2) routine clinical EEG examinations indicating slow, biphasic, and high‐voltage, centrotemporal sharp spikes, which are often followed by a slow wave; (3) normal brain MRI; (4) no other serious neurological, psychiatric, or somatic diseases (e.g., brain trauma, schizophrenia, autism spectrum disorder); (5) parents had normal intelligence, with a high school education or above, and children were receiving formal education. Notably, although the non‐IED group did not show IED during MEG, they showed typical centrotemporal sharp spikes during previous long‐term EEG (including sleep period) examinations and were diagnosed with SeLECTS by neurologists considering the medical history before enrollment. The exclusion criteria were as follows: (1) patients with parent‐reported learning disabilities; (2) contraindications on MRI or MEG; (3) patients who experienced seizures 3 days before the MEG scan; (4) patients with head movements of more than 5 mm during image acquisition. Children and their parents were informed of the purpose and procedure of the study and signed written informed consent was obtained. Ethics approval was obtained from the Nanjing Brain Hospital Ethics Committee (Nanjing, P. R. China).

**TABLE 1:** Clinical patients' data

Patients	Sex	Age (y)	Epilepsy duration (m)	Number of seizures	ASMs	Location of epileptic spikes (L, R, both)	Number of IED
1	F	8.3	0.6	1	None	R	8
2	F	9.4	12.5	2	None	L	8
3	M	6.4	2.8	4	None	L	11
4	F	9.4	0.4	2	None	L	9
5	F	9.7	2.6	2	None	L	12
6	M	8.0	3.0	3	VPA	R	10
7	M	9.1	11.9	3	OXC	both	10
8	F	7.7	2.2	1	None	both	8
9	F	10.0	2.5	2	OXC	L	9
10	F	8.4	0.3	1	None	both	11
11	M	7.1	8.0	2	None	L	9
12	M	7.1	1.1	2	None	R	10
13	F	10.4	2.2	4	None	R	10
14	F	8.0	13.0	15	LEV	R	12
15	M	10.0	12.0	3	None	R	11
16	F	4.8	1.0	1	None	–	
17	M	9.3	10.0	2	None	–	
18	M	10.3	1.0	1	None	–	
19	F	7.2	12.0	6	None	–	
20	M	9.2	1.1	2	None	–	
21	F	9.2	23.5	4	None	–	
22	F	8.3	4.4	3	None	–	
23	F	10.1	5.5	6	LEV	–	
24	F	10.2	7.7	4	None	–	
25	M	7.1	3.0	1	None	–	
26	F	6.4	8.8	7	None	–	
27	M	7.0	17.0	5	None	–	
28	F	6.9	2.4	6	LEV	–	
29	F	9.5	3.0	2	LEV	–	
30	M	10.0	6.0	2	None	–	

ASMs, antiseizure medications; L, left; LEV, levetiracetam; m, months; M/F, male/female;OXC, oxcarbazepine; R, right.; VPA, valproic acid; y, years.

### 2.2 Neurocognitive assessment

We used the Wechsler Intelligence Scale for Children, fourth edition (WISC‐IV) to measure intelligence of the subjects (Watkins & Smith, 2013). The results of the test included one full‐scale intelligence quotient (FSIQ) and four indices. The four indices are as follows: the Verbal Comprehension Index (VCI), the Perceptual Reasoning Index (PRI), the Working Memory Index (WMI), and the Processing Speed Index (PSI). Previous studies assumed that patients with SeLECTS showed impairment in various dimensions of cognitive function. The WISC‐IV was utilized in the present study to determine whether IED would cause a change in cognitive function. Previous studies chose an FSIQ score of 80 as the cutoff for cognitive decline, and in our study, the FSIQ scores of every participant were above 80.

### 2.3 MEG recording

MEG signals were collected in a magnetic‐shielded room using a whole‐head 275‐channel MEG system (VSM MedTech Systems, Inc., Coquitlam, BC, Canada) at the MEG Center of Nanjing Brain Hospital (Nanjing, P. R. China). Patients with SeLECTS were seizure‐free for 3 days before data collection and were asked to reduce their sleep time to increase the presence of IED. During data collection, subjects were asked to stay awake, remain quiet, close their eyes, relax their muscles, think of nothing, and avoid head movements. To monitor the position of the subject's head relative to the MEG sensors, three small electromagnetic coils were glued on the nasion and bilateral preauricular points of each subject. The MEG sampling frequency was 6000 Hz, and for each subject, at least six 120‐s sessions of MEG data were collected. Noise cancellation of third‐order gradients was performed on all recorded data. Data from subjects with movements of the head position of more than approximately 5 mm were excluded. To identify system and environmental noise, empty‐room MEG recordings were routinely completed before the experiment.

### 2.4 MRI scan

All subjects underwent MRI scans using a 3.0T MRI scanner (Siemens, Germany) after MEG recording to obtain structural T1 images. The T1‐weighted images were obtained using the following parameters: sagittal orientation, slices = 176, thickness = 1 mm, TE = 2.48 ms, TR = 1900 ms, matrix = 512 × 512, and field of view = 250 × 250 mm. Three small coils were placed in the nasion and preauricular points of the participants before scanning—the same places used in MEG recording to simultaneously register MRI and MEG data.

### 2.5 Data preprocessing

Interfering segments were removed by visual observation, and IEDs were identified by two experienced MEG professionals. To ensure data stability, data from the IED group, the non‐IED group, and the HC group were collected for a 30‐s period. The fragments selected by the IED group contained about 10 IEDs for each participant. Data were analyzed in the following six frequency bands: δ (1−4 Hz), θ (4−8 Hz), α (8−12 Hz), β (12−30 Hz), γ (30−80 Hz), and ripple (80−250 Hz). Additionally, corresponding filtering was used before data analysis to avoid interference of the ambient alternating current power around the 50 Hz band. Finally, MEG data were calculated using the MEG processor (https://sites.google.com/site/braincloudx/).

### 2.6 FC analysis

Wavelet‐based beamformer technology was used to project the collected MEG signals from the sensor level to the source level, according to the procedures and algorithms described in previous reports (Miao et al., 2019; Wu et al., 2017a; Zhang et al., 2021). First, we used accumulated source imaging to localize the whole brain's significant neuromagnetic signals. Accumulated source imaging is an imaging technique that provides the volumetric reconstruction of the epileptic source activity over multiple frequency ranges (Xiang et al., 2015). Next, the neural network was constructed by calculating the signal correlation of two source pairs in the 30‐s time window. A correlation factor was calculated to analyze the relationship between the virtual sensor signals from the dual‐source pair according to the following formula:

(1) $$R_{\left(X_{a},X_{b}\right)}=\frac{C\left(X_{a},X_{b}\right)}{SX_{a}SX_{b}},\;$$

where $R_{\left(X_{a},X_{b}\right)}$ represents the correlation between the pair of magnetic sources “a” and “b,” and $X_{a}$ and$\;X_{b}$ represent the signals from two of the magnetic sources calculated in a pair. $C(X_{a},X_{b})$ represents the average signal of the two magnetic sources, while $SX_{a}$ and $SX_{b}$ are the standard deviations of the signals of the two magnetic sources (Wu et al., 2017b). A threshold was used as a checkpoint to ensure the quality of the data. For all source pairs, *T*‐values were calculated to determine the threshold of the connections, according to the following formula:

(2) $$T_{p}=\;R\sqrt{\frac{K-2}{1-R^{2}},}$$

where $T_{p}$ is the *T*‐value of a correlation; *R* is the correlation of a source pair, and *K* is the number of data points connected. In this study, a threshold of a $T_{p}$ value with a corresponding *p*‐value < .05 was used to obtain the FC networks (Wu et al., 2017b; Xiang et al., 2014). The FC distribution of each voxel‐based virtual sensor was collectively co‐registered to the MRI of each participant. In MRI views, blue represented inhibitory connections, and red represented excitatory connections.

### 2.7 GT analysis

GT analysis was used to detect the topological pattern of the brain network (Reijneveld et al., 2007). A graph consists of a finite set of nodes and edges, where a node is the object of the study, and an edge represents the relationship between two nodes (Rubinov & Sporns, 2010). In our study, the magnetic source in the network was the node, and the functional connection was the line connecting the nodes (Kanemura et al., 2011). The degree (*D*), strength (*S*), path length (*L*), and clustering coefficient (*C*) were calculated for each source pair to precisely define and compare network properties.

#### 2.7.1 Degree

The network degree (*D*) denotes the number of connections between a node and other nodes, thereby reflecting the importance of the node (Reijneveld et al., 2007). In a weighted network, *G* containing *N* nodes and *K* edges, the network degree ($D_{A}$) is the average of the degrees of all nodes in the network:

(3) $$D_{A}=\frac{1}{N}\;\sum_{i\;=\;1}^{N}d_{i}.$$

In Equation (3), $D_{A}$ is an important marker of network development and compliance (Rubinov & Sporns, 2010).

#### 2.7.2 Strength

In a weighted network *G*, the connection strength (*S_i_*) of node “*i*” is defined as the sum of the weight values of the edges that are directly connected to it (Reijneveld et al., 2007):

(4) $$S_{i}=\frac{1}{N}\;\sum_{i\;=\;1}^{N}w_{ij},$$

while the strength of the network ($S_{A}$) refers to the average of the connection strength of all nodes:

(5) $$S_{A}=\frac{1}{N}\;\sum_{i\;=\;1}^{N}S_{i}.$$

#### 2.7.3 Path length

The path length between two nodes “i” and “j” refers to the sum of the lengths of the edges along the path connecting these two points. The length of each edge is equal to the inverse of the weight value of the edge, namely 1/*w_ij_*_._ The average path length ($L_{A}$) of the network is the average value of the path length of all node pairs in the network:

(6) $$L_{A}=\frac{1}{N\left(N-1\right)}\sum_{i,j}L_{i,j}.$$

In Equation (6), $L_{i,j}$ is the shortest distance between node *i* and *j*. This definition assumes that $L_{i,j}$= 0 if node *i* cannot be reached by node *j* or if *i* = *j*. *L_A_* reveals the global structure of the network.

#### 2.7.4 Clustering coefficient

The clustering coefficient was obtained to measure the local connectivity (Adebimpe et al., 2015). The definition is as follows: if a node sends *k* edges, the possible maximum number of edges that can exist between the nodes (*k*) connected by these *k* edges is *k* (*k* − 1)/2. The clustering coefficient of this node is defined as the score value obtained by dividing the actual number of edges by the maximum number of edges (Reijneveld et al., 2007). For a weighted network *G*, containing *N* nodes and *K* edges, the clustering coefficients of node *i* is determined as the average geometric weight of all nodes:

(7) $$C_{i}=\frac{1}{d_{i}\left(d_{i}-1\right)}\;\sum_{j,k\in Gj,k\neq i}{\left(w_{ij}\cdot w_{jk}\cdot w_{ki}\right)}^{1/3}.$$

In Equation (7), *d_i_* represents the number of edges that the node *i* is directly connected to (the degree of the node *i*), and *w* refers to the weight of the edges that connect the two nodes. The clustering coefficient ($C_{A}$) of network *G* is defined as the average of the clustering coefficient of all nodes in the network, and reflects the local characteristics of the network:

(8) $$C_{A}=\frac{1}{N}\;\sum_{i\;=\;1}^{N}C_{i}.$$

### 2.8 Statistical analysis

Statistical analysis was performed using SPSS software package version 26.0 (IBM Inc., Chicago, IL, USA). The differences in neural FC network patterns between the IED group, the non‐IED group, and the HC group were analyzed using the Fisher exact probability method. The Shapiro–Wilk test was used to assess the normality of the data, and a homogeneity test of variance was conducted. One‐way ANOVA was used to compare data when both normality and variance chi‐square were satisfied, while the Kruskal–Wallis *H*‐test was used when they were not satisfied. Spearman or Pearson correlation analysis was used to analyze the correlation between the clinical characteristics, the neurocognitive scores, and the graph theory (GT) parameters of each frequency band. The threshold of statistical significance was set at *p* < .05. Bonferroni multiple comparison was used to obtain the corrected *p* values of the data between the three groups (e.g., for three groups, *p* < .016).

## 3 RESULTS

### 3.1 Subjects

In this study, 30 children with SeLECTS and 15 healthy children were included. The IED group (15 patients) included six males, had a mean age of 8.6 ± 1.2 years, a mean disease duration of 5.0 ± 4.9 months, and an average of 3.1 ± 3.4 seizures. The non‐IED group (15 patients) included six males, had a mean age of 8.3 ± 1.7 years, a mean disease duration of 7.1 ± 6.4 months, and an average of 3.5 ± 2.1 seizures. The HC group, which included five males, had a mean age of 8.5 ± 2.1 years. The statistical analysis showed no significant differences between the three groups in terms of sex and age. Meanwhile, though the non‐IED group had a longer disease duration and more seizures than the IED group, there was no statistical difference between these data. Clinical patients' data are listed in Table 1.

### 3.2 WISC‐IV scores

The IED group had lower FSIQ, VCI, and WMI scores compared to the HC group and a lower VCI compared to the non‐IED group. In addition, the non‐IED group has a lower VCI compared to the HC group. Details of the performance for each group are provided in Table 2.

**TABLE 2:** WISC‐IV scores of IED group, non‐IED group, and healthy controls

	IED group	non‐IED group	HC group	*p*: IED vs. non‐IED	*p*: IED vs. HC	*p*: HC vs. non‐IED
FSIQ	95.53 ± 6.37	102.73 ± 11.49	111.67 ± 12.21	.191	<.001*	.069
VCI	87.53 ± 10.82	100.00 ± 14.56	114.53 ± 12.42	.031*	<.001*	.009*
PRI	105.06 ± 8.67	108.80 ± 13.26	108.87 ± 12.02	1.000	1.000	1.000
WMI	92.27 ± 8.79	99.33 ± 12.27	105.40 ± 10.29	.221	.004*	.368
PSI	103.67 ± 12.99	99.20 ± 10.11	101.73 ± 12.03	.914	1.000	1.000

FSIQ, Full‐Scale Intelligence Quotient; PSI, Processing Speed Index; PRI, Perceptual Reasoning Index; VCI, Verbal Comprehension Index; WMI, Working Memory Index;. Values are numbers or mean ± standard deviation (SD). *p < .05 after Bonferroni multiple comparisons.

### 3.3 FC networks

**FIGURE 1:**
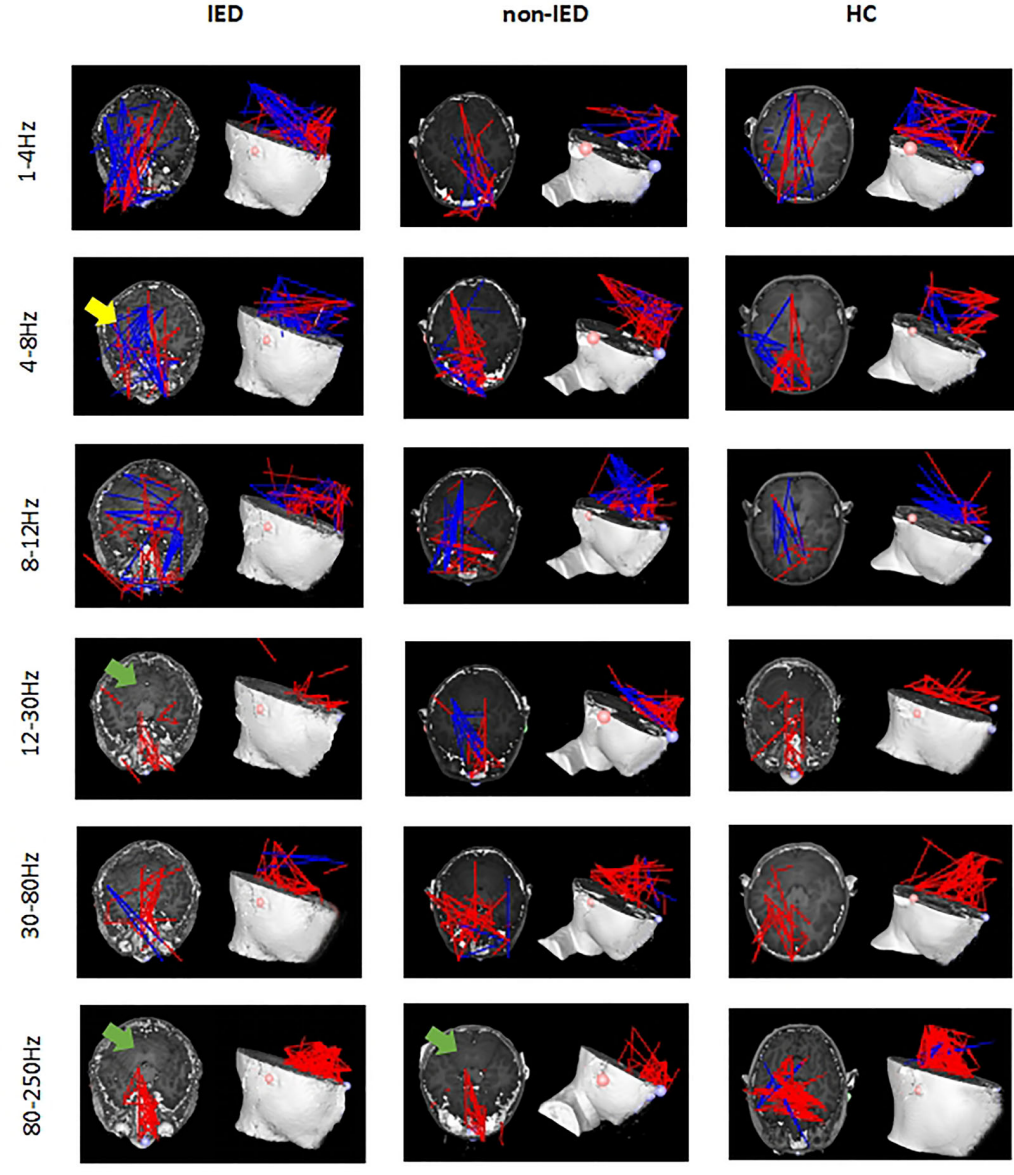
Typical FC network patterns in the 1– 250 Hz frequency range in the interictal epileptiform discharge (IED) group, the non‐IED group, and the HC group. The green arrow indicates a significant difference between anterior and posterior brain regions. The yellow arrow indicates a more dispersed whole‐brain network distribution pattern.

Our MEG results showed that the IED group had more dispersed FC in the 4–8 Hz frequency band, and more brain regions were involved compared to the other two groups. Furthermore, the IED group had fewer FC between the anterior and posterior brain regions in the 12–30 Hz frequency band. Both the IED group and the non‐IED group had fewer FC between the anterior and posterior brain regions in the 80–250 Hz frequency band compared to the HC group. In other frequency bands, no significant differences were observed. The FC networks between the three groups are shown in Figure 1.

### 3.4 GT analysis

**FIGURE 2:**
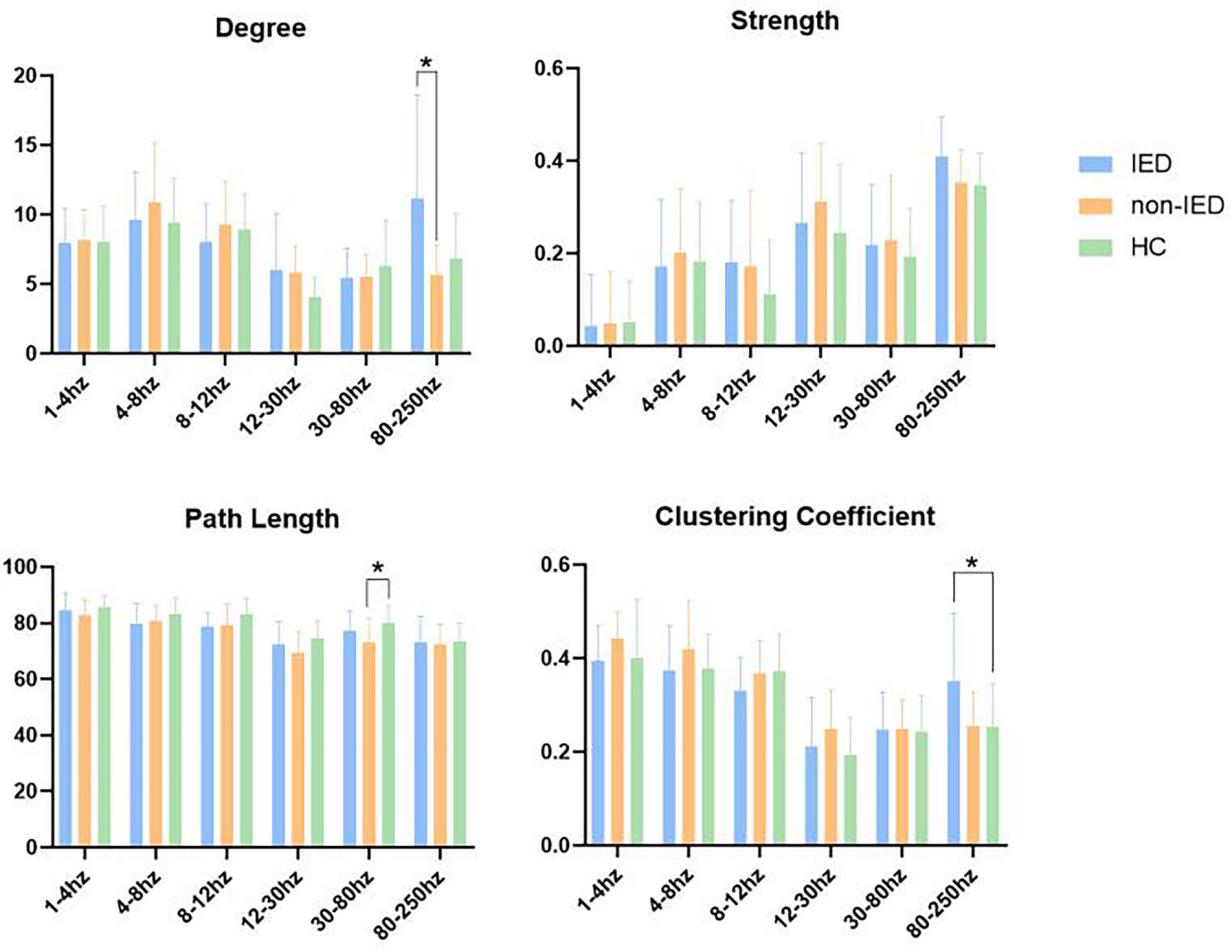
Differences in GT parameters (degree, strength, path length, and clustering coefficient) between the interictal epileptiform discharge (IED) group, the non‐IED group, and HC group. *p < .05 after Bonferroni multiple comparisons.

GT analysis showed that the IED group had a higher clustering coefficient (*p* = .047) compared to the HC group and a higher degree (*p* = .011) compared to the non‐IED group in the 80–250 Hz frequency band. The non‐IED group had a lower path length (*p* = .035) in the 30–80 Hz frequency band compared to the HC group. No significant differences were observed in other frequency bands. The GT values in the three groups in the six frequency bands are shown in Figure 2.

### 3.5 Correlation analysis

**FIGURE 3:**
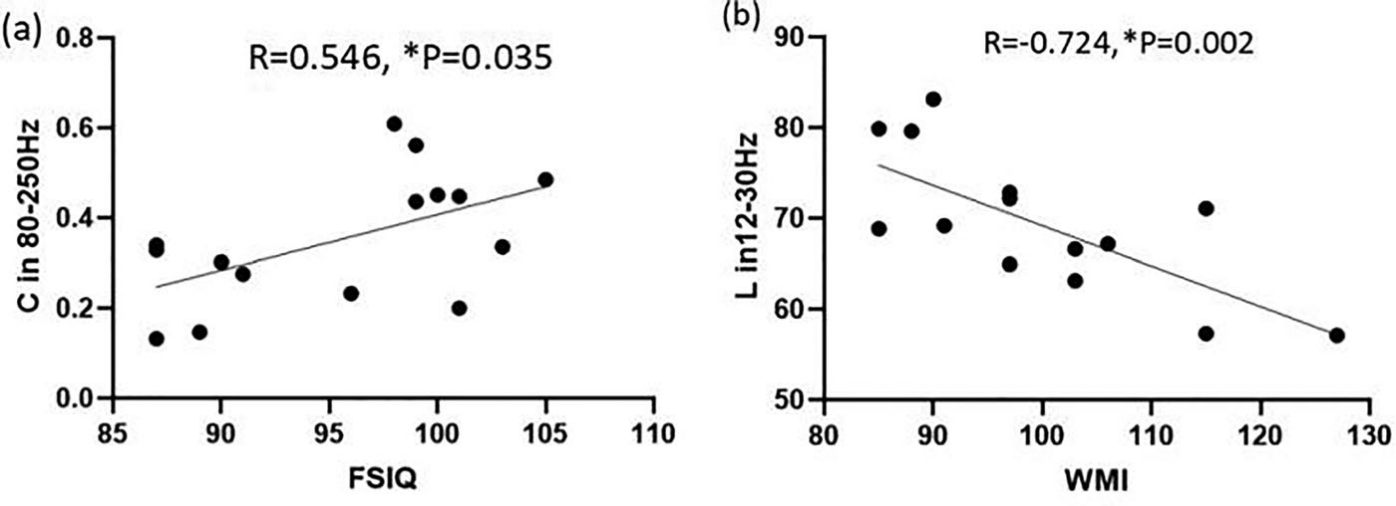
(a) Showed that the FSIQ in the IED group was positively associated with *C* in the 80–250 Hz frequency band. (b) Showed the WMI in the non‐IED group was negatively associated with *L* in the 12–30 Hz frequency band. * *p* < .05 after Bonferroni multiple comparisons.

The FSIQ in the IED group was positively associated with *C* in the 80–250 Hz frequency band (*R* = 0.546, *p* = .035). The WMI in the non‐IED group was negatively associated with *L* in the 12–30 Hz frequency band (*R* = −0.724, *p* = .002). No other significant correlations were obtained between the GT parameters and WISC‐IV scores. The results of this study did not indicate a significant correlation between GT parameters (degree, strength, path length, and clustering coefficient) and age, epilepsy duration and seizure frequency in the IED group and the non‐IED group. The result of the correlation analysis is shown in Figure 3.

## 4 DISCUSSION

In the normal brain, excitatory and inhibitory circuits interact to maintain a dynamic balance. This homeostasis is critical in the transmission of information (Turrigiano, 2011). Abnormal neuronal discharge may disrupt the homeostatic system in the brain and interfere with normal brain neural networks, thus, causing neurological dysfunction (Dai et al., 2022; Xiao et al., 2016). Growing evidence has suggested that SeLECTS is not a benign disease, since some children show attention deficits at varying degrees, as well as language dysfunction and cognitive decline (Xiao et al., 2015). Many studies now regard IED as a potential mechanism by which epilepsy disturbs the normal organization of brain networks and thereby leads to abnormal cognitive function (Li et al., 2018). In this study, a whole‐brain network was constructed to investigate the differences between the IED group, the non‐IED group, and HC group regarding network patterns and network topology. The correlation between GT parameters and WISC‐IV scores, age, epilepsy duration, and seizure frequency in the IED group and the non‐IED group was also investigated.

### 4.1 Neuropsychological test results

Our study showed that the IED group had a lower FSIQ compared to the HC group. It was previously reported that children with SeLECTS had early cognitive impairment (Li et al., 2020). Childhood is a critical period for brain development, and a growing evidence revealed that frequent IED can affect the formation and maturation of neural circuits, resulting in cognitive impairment (Nissenkorn et al., 2017). In addition, the IED group had lower VCI and WMI scores and the non‐IED group had lower VCI compared to the HC group. A study found that SeLECTS (SWI < 50%) patients and SeLECTS (SWI ≥ 50%) scored significantly lower than HCs on both the VCI and WMI, which was consistent with our results (Li et al., 2022). VCI represents language and verbal skills, Xiao et al. (2016) suggested that IED directly disrupt the functional brain networks responsible for language and behavior in SeLECTS patients. This may partially explain the impairment in the language dimension that patients present with. WMI reflects working memory. Different sleep stages are associated with the consolidation of specific memory systems, whereas IED occurs mainly during slow‐wave sleep, and may interfere in the dialogue between the temporal and frontal cortex, causing memory deficits (Verrotti et al., 2014). Li et al. (2022) also assumed that the higher the number of discharges during the interictal period, the greater the impact on cognitive function. This may help to explain why the IED group scored significantly lower compared to the non‐IED group on VCI in our study.

### 4.2 Network pattern

In this study, we found significant differences in network patterns between the three groups in the 4–8, 12–30, and 80–250 Hz frequency bands. The data showed that the FC network was confined to the frontal lobe in the IED group in the 12–30 Hz frequency band compared to the other two groups. Meanwhile, both the IED group and the non‐IED group had fewer FC between the anterior and posterior brain regions in the 80–250 Hz frequency band compared to the HC group.

This reflected enhanced connectivity in local cortical areas and weakened connectivity between anterior and posterior brain regions. Sun et al. (2021a) reported the localized pattern of FC networks in a childhood absence epilepsy study and considered that hypersynchronized FC networks involving the frontal cortex were a critical factor in the onset of absence seizures. It is hypothesized that the increase in short‐range synchronization among local neurons along with the decrease in long‐range synchronization between distant brain regions might promote the generation of epileptic discharges (Kramer & Cash, 2012; Sobayo et al., 2013). The brain network distribution structure during adolescence tends to be mature and becomes more specialized in function (Moeller et al., 2013). The reduced FC between the anterior and posterior cortices may reflect the immaturity of brain development. EEG analysis showed functional network reorganization in the frontal regions of patients with SeLECTS regardless of the presence of spikes in the EEG (Adebimpe et al., 2015). Considering that the frontal lobes are responsible for many higher‐order cognitive functions (Smallwood et al., 2021), the abnormal network patterns observed in this study may help explain cognitive impairment in children with SeLECTS. A longitudinal study by Niu et al. (2021) on the effects of antiseizure medications (ASMs) on the cognitive function of children with SeLECTS showed that patients demonstrated a reduced FC between anterior and posterior brain regions before treatment. Moreover, they observed enhanced FC between anterior and posterior brain regions and an improved cognitive function score in patients after 1‐year of treatment with ASMs and hypothesized that the normalization of FC in children with SeLECTS after treatment was likely the reason for improved cognitive function (Niu et al., 2021).

The FC of the IED group involved more brain regions in the 4–8 Hz band compared to the other two groups. Epilepsy is caused by increased abnormal synchronization of the brain neurons (Ibrahim et al., 2012). It was previously thought that epileptiform activity arose in the Rolandic area (Masterton et al., 2013). However, increasing evidence has suggested that changes in the network are not limited to the epileptogenic area, but also involve places far from this area. In a previous MEG study, it showed that children with benign epilepsy with bilateral centrotemporal spikes have significantly distracted connections in their brain regions (Wang et al., 2021). Beyond the seizure onset zone, the propagation of epileptiform discharges through an underlying network can lead to a secondary pathology of distal cortical regions and subcortical regions, which may explain the increased connectivity in other areas of the IED group (Lin et al., 2012).

In the present study, it revealed that network changes were frequency‐dependent. Each frequency band in the human brain has physiological significance and is associated with specific processes (Sauseng & Klimesch, 2008), which could partially explain the variation in FC network patterns observed in specific (but not all) frequency bands.

Networks of different sizes oscillate at specific frequencies. Theta oscillations are associated with emotional responses (Knyazev, 2007). Beta oscillations are considered to play an important role in attention (Sauseng & Klimesch, 2008). High‐frequency oscillations (>80 Hz) are suitable for detecting adjacent and strongly connected neural connections, and for better detecting neuronal discharge in specific focal regions, now often used for the localization of epileptogenic areas (Engel & da Silva, 2012; Sun et al., 2020b).

The findings of this study showed differences in network patterns in these bands between the three groups, but the specific physiological mechanism underlying network changes in each band needs to be further explored.

### 4.3 GT analysis

Differences in network topology between the IED group, the non‐IED group, and the HC group in different frequency bands were observed using GT analysis. Although the non‐IED group did not show IED during data collection, a change in the neural network due to chronic epileptogenic processes cannot be excluded. *C* measures the network separation and *L* measures the network integration (Rubinov & Sporns, 2010). Normal brain functional networks have small‐world properties (higher *C* and lower *L*) since brain networks combine the respective topological advantages of regular networks (high *L*, high *C*) and random networks (short *L*, short *C*) (Zhang et al., 2011). This allows the process of information to be more efficient by the human brain while saving wiring costs. The data showed that *L* decreased in the non‐IED group in the 30–80 Hz frequency band compared to the HC group, which indicated that the network tended to be more randomized and had decreased efficiency in the local information transfer. It is suggested that randomized networks have better synchronization compared to normal networks, thus, facilitating the occurrence of IED (Adebimpe et al., 2015). In addition, randomized networks have reduced abilities for information processing and fault tolerance (Xiao et al., 2015). A study performed by Ji et al. (2017). showed reduced global and local efficiency (decreased global and regional efficiency) in patients with SeLECTS. The pathological network of short *C* and short *L* has also been reported in previous studies including patients with temporal lobe epilepsy (Liao et al., 2010). The IED group had a higher *C* compared to the HC group in the 80–250 Hz frequency band. This may represent that the brain functional networks of the IED group show more orderly organization at certain frequency band. Thus, we hypothesized that IED might disturb the normal network topology and disrupt the small‐world properties of the normal neural networks. The ability of the human brain to dynamically adjust the network structure under environmental changes explains why humans can generate complex cognitive functions (Cohen & D'Esposito, 2016). The weaker small‐world properties and reduced ability to separate and integrate neural networks in the IED group and the non‐IED group could partially contribute to explaining the reduced cognitive level in SeLECTS patients. *H* was higher in the IED group compared with the non‐IED group in the 80–250 Hz frequency band, thereby indicating that the IED group had higher network integration. This suggested the overconnectivity of brain networks, potentially leading to ineffective information processing. During the development and maturation of the human brain, neural circuits in the central nervous system are continuously remodeled and eventually form mature neural circuits that regulate higher‐order cognitive functions and neural activities. This process relies on synaptic pruning, which means selectively eliminating redundant synapses and maintaining, strengthening and refining the remaining synapses. Synaptic pruning is essential for the formation of mature neural circuits (Overvliet et al., 2013). Frequent IED can affect synaptic activity and pruning, which may lead to redundant connections, affect the dynamic development of the brain, and interfere with the maturation process of normal neural circuits (Bourel‐Ponchel et al., 2019; Overvliet et al., 2013). The data obtained in this study showed a variation of GT parameters in the high‐frequency band. Considering there are few reports on high‐frequency signals, our study can serve as a complement to the network of high‐frequency neural signals in the brain.

In conclusion, the data in this study showed changes in network patterns and network topology in specific frequency bands in the IED group and the non‐IED group, thereby suggesting that the epileptogenic process might interfere with the normal network. Although IED were only transient states, the chronic epileptogenic process may cause irreversible changes in the brain.

### 4.4 Correlation analysis

Our study showed that the FSIQ in the IED group was positively associated with *C* in the 80–250 Hz frequency band and the WMI in the non‐IED group was negatively associated with *L* in the 12–30 Hz frequency band. Previous MEG studies reported that better cognitive performance was related to higher *C* and lower *L* values in certain frequency bands, which was similar with our results (Li et al., 2020; Wang et al., 2021). Normal brain functional networks have higher *C* and lower *L* within a certain range, which can make the brain more efficient in information integration and processing (Zhang et al., 2011). These results further suggested that alterations in the topological pattern of the brain network may be the mechanisms through which cognitive impairment occurs in patients with SeLECTS.

This study did not show a correlation between network parameters and age, epilepsy duration, and seizure frequency in the IED group and the non‐IED group. We supposed that some limitations were responsible for this negative result. First, the size of the sample was small. Second, the age onset of patients might be earlier than that mentioned by their parents, thus, the number and duration of onset might not be accurately provided. These problems are difficult to resolve, and in future studies, more patients will be included, and participants with unclear clinical data will be excluded as much as possible.

## 5 LIMITATIONS

This study has several limitations. First, the number of participants in the study was relatively small, and the ASMs taken by some patients may influence resting‐state brain networks. A larger sample size may enhance the generalizability of the results. Second, simultaneous EEG recordings during the MEG scan were not performed. Third, artifacts from electromyography, magnetocardiography, and other signals might influence the results although MEG signals were recorded under the same experimental conditions. Fourth, the effect of IED on resting‐state networks might be averaged out by the segments without IED for the long‐time segment selected by the IED group. In future studies, more neuropsychological tests to accurately analyze the effect of IED on cognitive function should be performed.

## 6 CONCLUSIONS

In our study, the differences on resting‐state brain networks between the IED group, the non‐IED group, and the HC group were investigated using multifrequency MEG. In the IED group and the non‐IED group, small‐world properties deviated from normal state and the network patterns changed. Moreover, network patterns localized in the frontal lobe and fragmented FC found in specific frequency bands might help explain cognitive dysfunction in patients with SeLECTS. However, the present study was a preliminary study and the effect of IED on cognitive function and brain networks should be further analyzed in the future using a larger sample size and more neuropsychological scales.

## CONFLICT OF INTEREST

The authors of this study declare that they have no conflict of interest.

### PEER REVIEW

The peer review history for this article is available at https://publons.com/publon/10.1002/brb3.3038.

## Data Availability

The data that support the findings of this study are available on request from the corresponding author. The data are not publicly available due to privacy or ethical restrictions.
